# Pyridinyl-Carbazole Fragments Containing Host Materials for Efficient Green and Blue Phosphorescent OLEDs

**DOI:** 10.3390/molecules26154615

**Published:** 2021-07-30

**Authors:** Dovydas Blazevicius, Daiva Tavgeniene, Simona Sutkuviene, Ernestas Zaleckas, Ming-Ruei Jiang, Sujith Sudheendran Swayamprabha, Rohit Ashok Kumar Yadav, Jwo-Huei Jou, Saulius Grigalevicius

**Affiliations:** 1 Department of Polymer Chemistry and Technology, Kaunas University of Technology, Radvilenu Plentas 19, LT50254 Kaunas, Lithuania; dovydas.blazevicius@ktu.lt (D.B.); daiva.tavgeniene@ktu.lt (D.T.); 2 Department of Biochemistry, Faculty of Natural Sciences, Vytautas Magnus University, Vileikos Str. 8, LT44404 Kaunas, Lithuania; Simona.Sutkuviene@vdu.lt; 3 Institute of Agricultural Engineering and Safety, Agriculture Academy, Vytautas Magnus University, Studentu Str. 15A-212, Akademija, LT53362 Kauno Distr., Lithuania; zerniukas@yahoo.co.uk; 4 Department of Materials Science and Engineering, National Tsing-Hua University, No. 101, Kaungfu Rd. Hsin-Chu 30013, Taiwan; g8ai5up@gmail.com (M.-R.J.); sujithsudheendran.s.s@gmail.com (S.S.S.); rohitakyadav@gmail.com (R.A.K.Y.)

**Keywords:** carbazole, triplet energy, amorphous film, current efficiency, host material, emitter

## Abstract

Pyridinyl-carbazole fragments containing low molar mass compounds as host derivatives **H1** and **H2** were synthesized, investigated, and used for the preparation of electro-phosphorescent organic light-emitting devices (PhOLEDs). The materials demonstrated high stability against thermal decomposition with the decomposition temperatures of 361–386 °C and were suitable for the preparation of thin amorphous and homogeneous layers with very high values of glass transition temperatures of 127–139 °C. It was determined that triplet energy values of the derivatives are, correspondingly, 2.82 eV for the derivative **H1** and 2.81 eV for the host **H2**. The new derivatives were tested as hosts of emitting layers in blue, as well as in green phosphorescent OLEDs. The blue device with 15 wt.% of the iridium(III)[bis(4,6-difluorophenyl)-pyridinato-*N*,*C2*′]picolinate (FIrpic) emitter doping ratio in host material **H2** exhibited the best overall characteristics with a power efficiency of 24.9 lm/W, a current efficiency of 23.9 cd/A, and high value of 10.3% of external quantum efficiency at 100 cd/m^2^. The most efficient green PhOLED with 10 wt% of Ir(ppy)_3_ {tris(2-phenylpyridine)iridium(III)} in the **H2** host showed a power efficiency of 34.1 lm/W, current efficiency of 33.9 cd/A, and a high value of 9.4% for external quantum efficiency at a high brightness of 1000 cd/m^2^, which is required for lighting applications. These characteristics were obtained in non-optimized PhOLEDs under an ordinary laboratory atmosphere and could be improved in the optimization process. The results demonstrate that some of the new host materials are very promising components for the development of efficient phosphorescent devices.

## 1. Introduction

OLEDs (organic light-emitting devices) receive a lot of attention due to their growing applications in solid-state lighting devices, as well as in flat-panel display technologies [1]. The OLED-based displays are already demonstrated in the market starting from the last several years, and lighting technologies were also presented rapidly [2,3]. In the emerging technologies of organic electronics, phosphorescent organic light-emitting diodes (PhOLEDs) are among the most mature devices. In such a type of device, discovered in 1998, the emitting layer is constituted of a heavy-metal complex emitter dispersed within a host material in order to harvest both singlet and triplet excitons. This technique allows the PhOLED to reach an internal quantum efficiency (IQE) of 100%, whereas the IQE of a fluorescent OLED is only 25%. At this time, the phosphorescent derivatives are ideal for the production of the devices with high efficiency due to the exploitation of singlet and triplet excitons of the emitters simultaneously through intersystem crossing [4,5,6,7]. In the phosphorescent OLEDs (PhOLEDs), the mentioned triplet emitters in layers are dispersed as emitting guests in host materials to decrease quenching due to relatively long excited-state lifetimes of the triplet emitting materials and to reduce triplet-triplet annihilation. For these reasons, the required host derivatives are widely synthesized and investigated for the production of the PhOLEDs [8,9,10].

It is important for the efficient electro-phosphorescence that the triplet level energy of a used host derivative would be higher than that of the used triplet dopant to prevent reverse energy transmittance from the emitter back to the host and to confine as many as possible triplet excitons on guest structures [11,12,13]. Another requirement for the effective operation of the PhOLEDs is the ability of the host to create amorphous and stable layers with high values of the glass transition temperature. The film-forming property ensures that the guest molecules stay uniformly dispersed in the host material to decrease the possibility of concentration quenching.

It was described earlier that various compounds containing electronically isolated indolyl and/or carbazolyl substituents have rather large values of the triplet energy and are useful amorphous hosts for the PhOLEDs [14,15,16,17,18]. Here, we describe new well soluble pyridinyl-carbazole-based host materials for solution-processed emitting layers of the electro-phosphorescent technologies. The bipolar nature of the aromatic chromophore of the derivatives is responsible for bipolar charge transfer in the emitting layer. On the other hand, the oxetane unit in the structure affects the high glass transition temperature of the materials, as well as improved film-forming properties.

## 2. Experimental

### 2.1. Preparation of the Objective Materials

9H-carbazole (**1**), 3,3-bis(chlormethyl)oxetane, boronic acid of 2-methoxy-3-pyridine, 6-boronic acid of 6-methoxy-3-pyridine, KOH, K_2_CO_3_, butan-2-one, tetrahydrofuran (THF), and catalyst PdCl_2_(PPh_3_)_2_ were received from Aldrich and used without purification.

The 3-Iodo-9H-carbazole (**2**) was synthesized by using Tucker’s method [19].

The 3,3-Di(3-iodo-9-carbazolylmethyl)oxetane (**3**) was prepared by reaction of the 3,3- di(chloromethyl)oxetane and 3-iodo-9H-carbazole (**2**) by using the procedure we described earlier [20].

The 3,3-Bis [3-(2-methoxy-3-pyridinyl)carbazol-9-ylmethyl]oxetane (**H1**): 3,3-Bis(3-iodocarbazol-9-ylmethyl)oxetane **3** (0.8 g, 0.0012 mol), 2-methoxy-3-pyridinylboronic acid (0.46 g, 0.03 mol), potassium hydroxide (0.34 g, 0.006 mol), and catalyst PdCl_2_(PPh_3_)_2_ (0.034 g, 0.000048 mol) were stirred in mixture of THF (12 mL) and degassed water (1.5 mL) at reflux for 1 h. After the TLC test, the reaction mixture was cooled and filtered. Solvents were removed by evaporation. The objective product was separated by silica gel chromatography. A mixture of ethyl acetate and hexane (vol. ratio 1:7) was used as eluent. Yield: 0.32 g (27%) of yellow material. M.p.: 257 °C (DSC). ^1^H NMR (400 MHz, CDCl_3_, δ, ppm): 8.23 (s, 2H, Ar), 8.17 (t, 4H, Ar, J = 7.2 Hz), 7.71 (dd, 2H, Ar, J_1_ = 1.6 Hz, J_2_ = 7.2 Hz), 7.65 (dd, 2H, Ar, J_1_ = 1.4 Hz, J_2_ = 8.6 Hz), 7.46 (t, 2H, Ar, J = 7.6 Hz), 7.37–7.25 (m, 6H, Ar), 7.01 (m, 2H, Ar), 4.73 (s, 4H, -N-CH_2_-), 4.70 (s, 4H, -CH_2_-O-), 4.00 (s, 6H, CH_3_-O-). ^13^C NMR (400 MHz, CDCl_3_) δ 161.05, 145.25, 141.80, 140.88, 138.80, 130.96, 128.49, 127.54, 126.31, 125.18, 123.45, 121.35, 120.75, 119.96, 117.21, 108.84, 108.44, 75.93, 53.61, 50.74, 47.53. MS (APCI^+^, 20 V): 653.19 ([M + Na]^+^, 100%).

The 3,3-Bis [3-(4-methoxy-3-pyridinyl)carbazol-9-ylmethyl]oxetane (**H2**): 3,3-Bis(3-iodocarbazol-9-ylmethyl)oxetane **3** (0.8 g, 0.0012 mol), 6-methoxy-3-pyridinylboronic acid (0.46 g, 0.03 mol), potassium hydroxide (0.34 g, 0.006 mol) and catalyst PdCl_2_(PPh_3_)_2_ (0.034 g, 0.000048 mol) were stirred into a mixture of THF (12 mL) and degassed water (1.5 mL) at reflux for 1 h. After the TLC test, the reaction mixture was cooled and filtered. The solvent was evaporated and the objective product was separated by silica gel column chromatography using the mixture of ethyl acetate and hexane (vol. ratio 1:7) as an eluent. Yield: 0.23 g (20%) of yellow amorphous material. T_g_ = 139 °C (DSC). ^1^H NMR (400 MHz, CDCl_3_, δ, ppm): 8.47 (d, 2H, Ar, J = 2.4 Hz), 8.25 (d, 2H, Ar, J = 1.2 Hz), 8.17 (d, 2H, Ar, J = 7.6 Hz), 7.89 (dd, 2H, Ar, J_1_ = 2.4 Hz, J_2_ = 8.4 Hz), 7.59 (dd, 2H, Ar, J_1_ = 2 Hz, J_2_ = 8.4 Hz), 7.46 (t, 2H, Ar, J = 7.4 Hz), 7.37–7.28 (m, 6H, Ar), 6.85 (d, 2H, Ar, J = 8.8 Hz), 4.73 (s, 4H, -CH_2_-N-), 4.68 (s, 4H, -CH_2_-O-), 4.00 (s, 6H, CH_3_-O-). ^13^C NMR (400 MHz, CDCl_3_) δ 163.27, 144.97, 141.83, 140.82, 137.73, 130.71, 129.97, 126.52, 125.17, 124.00, 123.28, 120.77, 120.04, 118.74, 110.81, 109.18, 108.88, 75.94, 53.58, 50.76, 47.47. MS (APCI^+^, 20 V): 653.23 ([M + Na]^+^, 100%).

### 2.2. Instrumentation and Structures of the PhOLEDs

The Bruker Avance III (400 MHz) apparatus was used for recording ^1^H and ^13^C nuclear magnetic resonance (NMR) spectra. The data are provided as chemical shifts (δ) in ppm against trimethylsilane. The Waters ZQ 2000 mass spectrometer was utilized for recording mass spectra.

The TGAQ50 apparatus was used for TGA (thermo-gravimetric analyses). The Bruker Reflex II DSC apparatus was utilized for differential scanning calorimetry (DSC) measurements. The TGA and DSC measurements were performed at a heating rate of 10 °C/min in a nitrogen atmosphere.

**Figure 1:**
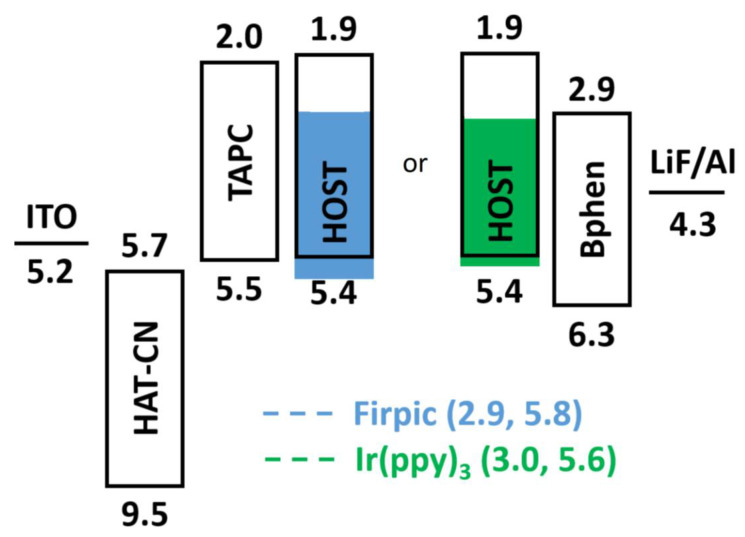
Structure and energy levels of blue and green PhOLEDs with host **H1**.

The PhOLED devices were fabricated on cleaned indium tin oxide (ITO) sputtered glass substrates. The soap solution, deionized water, acetone, and alcohol were used for cleaning the substrates, followed by ultraviolet ozone treatment. The energy levels and structure of blue and green devices using host **H1** are shown in Figure 1 as an example.

All the described OLEDs were formed from ITO (125 nm) as an anode, a layer of 1,4,5,8,9,11-hexaazatriphenylenehexacarbonitrile (HAT-CN, 30 nm) for hole injection, and a hole transporting layer of 1,1-bis[(di-4-tolylamino)phenyl]cyclohexane (TPC, 20 nm). The layers of HAT-CN and TPC were prepared by the thermal evaporation technique. A single emissive layer was used in all the OLEDs with different doping concentrations of phosphorescent blue FIrpic emitter or green Ir(ppy)_3_ emitter dispersed in the host materials **H1** or **H2**. The emitting layers of 50 nm were prepared by spin-coating from chloroform solutions (formed at 2000–2500 rpm for 20 s). The 4,7-Diphenyl-1,10-phenanthroline (Bphen, 20 nm) was thermally evaporated for the electron transporting layer. LiF (1 nm) was formed by evaporation as an electron injecting layer (EIL), and Al (100 nm) was evaporated as a cathode in structures of all the OLEDs, as demonstrated in Figure 1.

Characteristics of the prepared devices were registered at room temperature. The current–voltage (I–V) measurements were performed with the Keithley 2400 electrometer Minolta CS-100. A Photo Research PR-655 spectro-radiometer was utilized for measurements of luminance, chromaticity coordinates (CIE), and spectra of the electroluminescence.

As it could be seen from the energy levels presented in Figure 1, the HOMO and LUMO energy values of the new host materials **H1** and **H2** were 5.4 and 1.9 eV, respectively, and were very similar due to similar chemical structures of the synthesized materials. The energy levels are not exactly the same as that of the used dopants but are still suitable for the preparation of the host–quest systems in the emitting layers by using the blue FIrpic or green Ir(ppy)_3_ emitters.

## 3. Results and Discussions

**Scheme 1:**
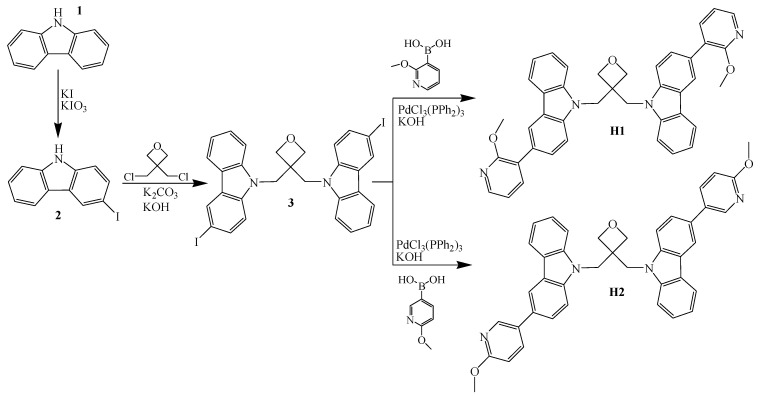
Synthetic pathway of materials **H1** and **H2**.

The objective host materials **H1** and **H2** were synthesized during the three synthesis steps, as shown in Scheme 1. The 3-Iodo-9H-carbazole (**2**) was firstly obtained by using the 9H-carbazole (**1**) iodination method of Tucker [19]. The 3,3-Di(3-iodo-9-carbazolylmethyl)oxetane (**3**), as a key material, was then made by the reaction of 3,3- di(chloromethyl)oxetane with an excess of the iodo-derivative **2** under basic conditions in the presence of the phase transfer catalyst—tetrabutylammonium hydrogensulfate. The objective compound **H1** was received by Suzuki reaction of the diiodo-compound **3** with an excess of 2-methoxy-3-pyridinylboronic acid. Derivative **H2** was also prepared by Suzuki reaction of the bi(3-iodocarbazole) derivative **3**. In this reaction, an excess of 6-methoxy-3-pyridinylboronic acid was used. All the prepared derivatives were confirmed by MS spectrometry, ^1^H, and ^13^C NMR spectroscopy. All these practically obtained data are in good agreement with theoretically developed structures.

The synthesized objective derivatives are well soluble in conventional organic solvents. The thermal evaporation technique could be used for the preparation of electroactive thin layers on substrates from these derivatives. The thin films could also be prepared by cheap spin coating from the solution method.

The behavior under thermal treatment of the synthesized objective host derivatives **H1** and **H2** was established by DSC and TGA. It was demonstrated that the derivatives obviously have high thermal stability. The temperature of 5% weight loss for the compounds **H1** and **H2** was 386 °C and 361 °C, respectively, as confirmed by TGA with a heating rate of 10 °C/min. It could be mentioned that the material with 2-methoxy-3-pyridinylcarbazole fragments has higher thermal stability than that of an analogous material with 4-methoxy-3-pyridinyl substituents. The TGA curves are presented as Supplementary Materials for the publication.

**Figure 2:**
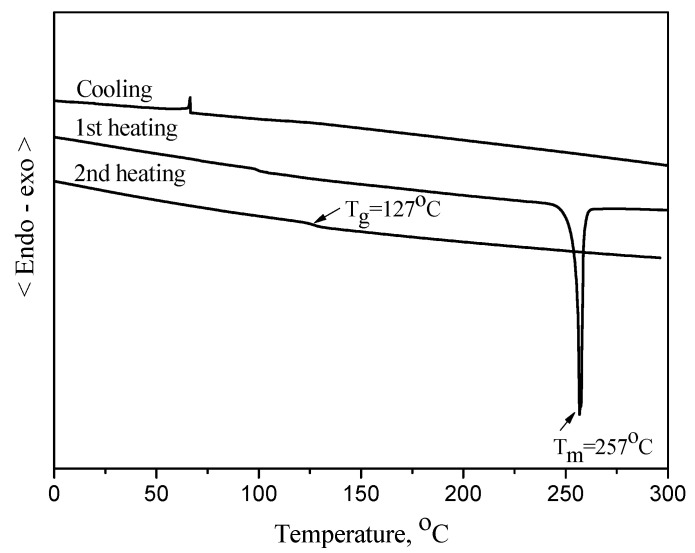
DSC curves of the material **H1**.

The derivative **H1** was obtained as crystalline material after its synthesis and purification by silica gel chromatography, as confirmed by DSC. During the first heating, the endothermic melting peak of the crystals was observed at 257 °C, as shown in Figure 2. When the melted sample was cooled down, it formed an amorphous material with a high glass transition temperature (T_g_) of 127 °C. Only the glass transition was also registered at the second heating of the material. No other signals due to melting or crystallization were observed by further heating of the used sample.

**Figure 3:**
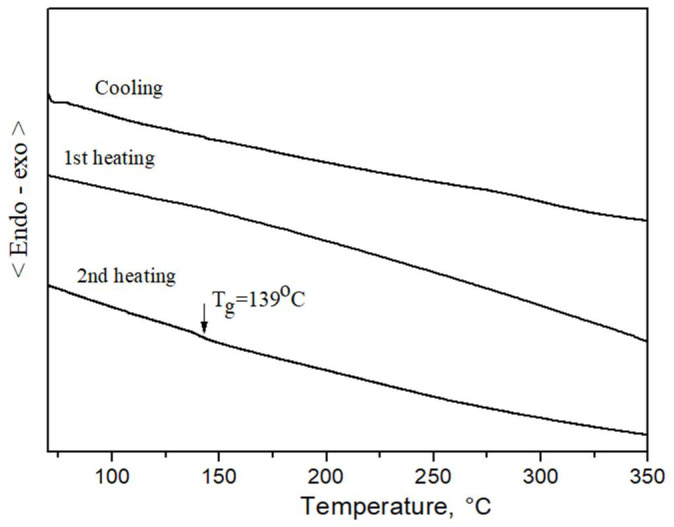
DSC curves of the derivative **H2**.

On the other hand, the compound **H2** was obtained as fully amorphous material after its synthesis, as confirmed by the DSC. The thermo-grams of the compound **H2** are shown in Figure 3. During the first and second heating scans, only the high glass transition temperature (T_g_) of 139 °C was detected for the compound. Crystallization or melting signals were not registered during these heating/cooling scans in the area from 0 °C to 350 °C.

Phosphorescent spectra, which were registered at 77 K, aimed to establish triplet energies of the host derivatives **H1** and **H2**. The spectra are presented as Supplementary Materials for the publication. It was calculated that values of the triplet energy are 2.82 eV for derivative **H1** and 2.81 eV for compound **H2**. The triplet energies of the hosts are higher than those declared for blue or green phosphorescent dopants, thus confirming their suitability as emitting hosts in green as well as in blue phosphorescent OLEDs applications.

**Figure 4:**
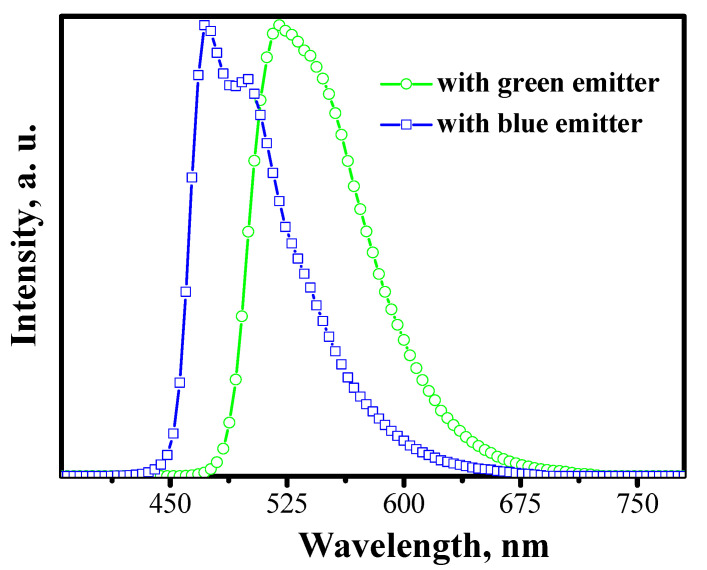
EL spectra of the OLEDs with host material **H1** with blue FIrpic or green Ir(ppy)_3_ dopants in emitting layers.

To demonstrate the performance of the prepared derivatives **H1** and **H2** as emitting hosts, green and blue PhOLEDs were formed by using blue FIrpic and also green Ir(ppy)_3_ phosphorescent emitters. Figure 4 demonstrates the electroluminescent (EL) spectra of the devices using the host material **H1**, which were registered at a brightness of 1000 cd/m^2^. As it could be seen from the Figure, the EL spectra of both the OLEDs confirmed pure emissions of, correspondingly, FIrpic or Ir(ppy)_3_ dopants, confirming the presence of suitable energy transfer between the host **H1** and the guests [21]. No additional emission zones were registered, demonstrating that the carrier recombination is located within the emitting layer and that the exciton diffusion to the hole or electron transporting layers is avoided in the OLEDs structures [22].

**Figure 5:**
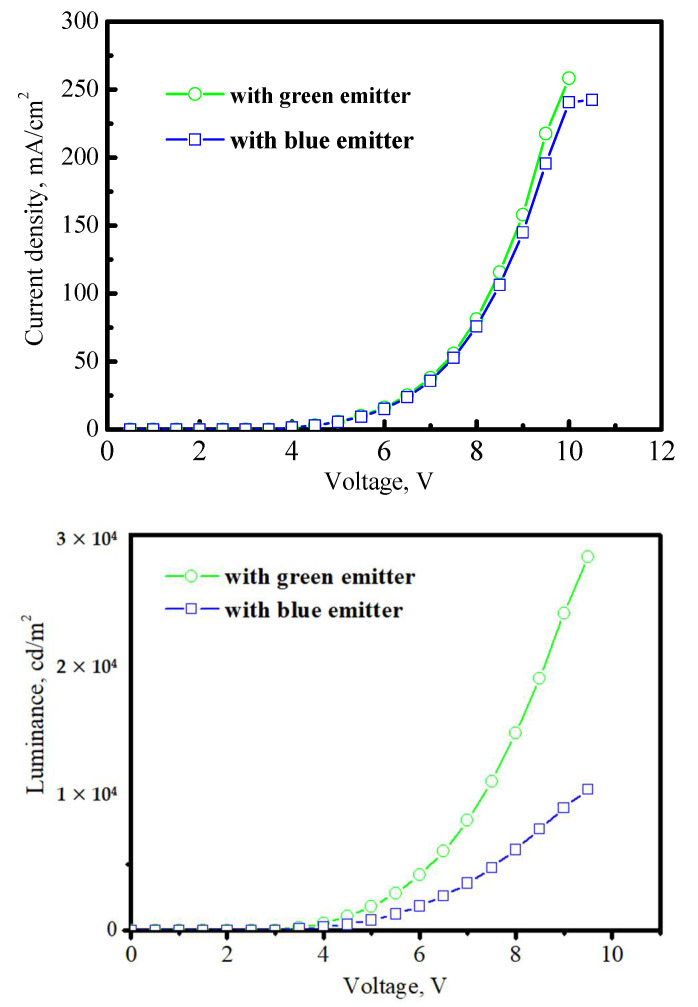

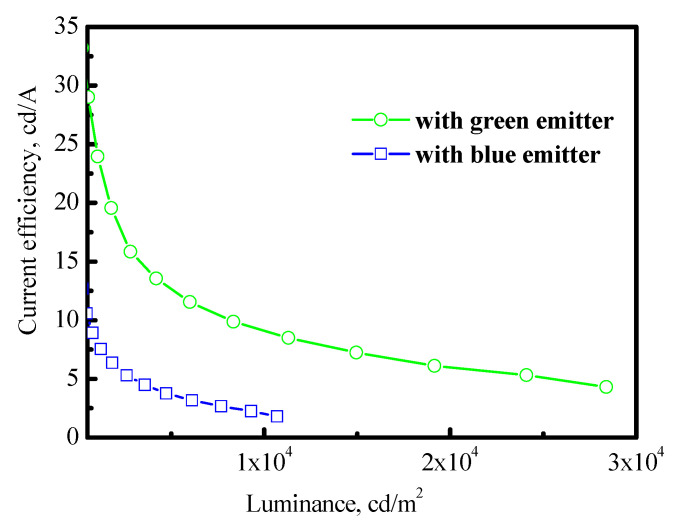
Characteristics of OLEDs with host **H1** with Ir(ppy)_3_ or FIrpic dopants in emitting layers.

The low molar mass host material **H1** was firstly tested in a concentration-dependent OLED formation with phosphorescent green Ir(ppy)_3_ dopant. The electro-phosphorescence of the devices was seen to originate only from the quest at different voltages in all the concentration-dependent OLEDs. No host and transporting layer compound molecular emission was observed from the devices, confirming the suitable energy transfer and/or charge transfer from the host material to the dopant and also the efficient transfer of holes as well as electrons into the emission layer. It was established that 12.5 wt.% of Ir(ppy)_3_ with green OLED demonstrated the best characteristics. Figure 5 presents the performance of the device. The green device showed a low turn-on voltage of 3 V, 49.8 cd/A of maximum current efficiency, maximum power efficiency of 33.2 lm/W, and 28,390 cd/m^2^ exceeding maximal brightness. At a higher brightness of 1000 cd/m^2^, which is required for illumination technologies, this device also demonstrated the highest efficiency of 34.6 cd/A (24.5 lm/W) between all the green OLEDs using host material **H1**.

To demonstrate the characteristics of the compound **H1** as a host of blue OLEDs, phosphorescent devices were formed by taking blue FIrpic dopant as the guest. The structure of the produced OLED is also described in the Experimental part. The material **H1** was also tested in concentration-dependent emitting layers. As it could be seen from Figure 5, the blue OLED with 20 wt.% of FIrpic as the dopant demonstrated the best overall characteristics with a low turn-on voltage of ca. 3 V, 18.3 cd/A of maximum current efficiency, maximum power efficiency of 12.7 cd/A, and 10,700 cd/m^2^ exceeding maximum brightness. The efficiency roll-off at a high current, which is typical for phosphorescent devices, was also seen here; however, at 1000 cd/m^2,^ brightness, a rather high efficiency of about 13.7 cd/A (8.3 lm/W) was still registered in the blue device.

The other host material, **H2**, was also applied in blue devices by using blue FIrpic dopant as the emitter. In order to optimize the concentration of the dopant in the emitting layer, **H2** was applied as the host in concentration-dependent experiments with the FIrpic amount ranging from 15 to 22.5 wt.%. Table 1 summarizes the OLED characteristics and efficiencies of the investigated PhOLEDs. It was observed that the OLED with 15 wt.% of the FIrpic doping ratio demonstrated the best characteristics with 23.9 cd/A of current efficiency, 24.9 lm/W of power efficiency, and high external quantum efficiency of 10.3% at a brightness of 100 cd/m^2^. At a higher brightness of 1000 cd/m^2^, which is desired for illumination technologies, this device also had the highest efficiency of 8.0% (18.4 cd/A, 15.8 lm/W) among all the blue devices using host **H2**. The maximum luminance of the OLED exceeded 9170 cd/m^2^.

The host material **H2** was also applied as a component of green PhOLEDs in concentration-dependent tests with the Ir(ppy)_3_ dopant amount in the range from 7.5 to 15 wt%. Table 1 summarizes the properties of the PhOLEDs. It can be mentioned that material **H2**-based green PhOLEDs had reasonable better performance than that of derivative **H1** using OLEDs. The devices with host **H2,** using dopant concentration ranging from 10 to 15 wt%., demonstrated rather low turn-on voltages of 2.6–3.3 V, power efficiencies of 26.2–34.1 lm/W, current efficiencies of 10.3–20.6 cd/A, and high external quantum efficiencies of 3.3–5.7% at a brightness of 100 cd/m^2^. As it could be observed, the 10 wt.% of Ir(ppy)_3_ containing OLED had the best characteristics with a current efficiency of 33.9 cd/A, power efficiency of 34.1 lm/W, and high external quantum efficiency of 9.4% at 1000 cd/m^2^ brightness, which is applied for illumination applications. The maximal brightness of the OLED reached almost 39,000 cd/m^2^.

It should be mentioned that these properties were demonstrated in non-optimized test PhOLEDs under an ordinary laboratory atmosphere. The PhOLEDs characteristics could be further modified by an optimization of the film thicknesses as well as layers formation conditions. It could be seen that the efficiency of all the presented devices decreases rapidly with the increase in luminance or current density. The phenomenon of organic light-emitting diodes is termed the efficiency roll-off. In particular, phosphorescent organic light-emitting diodes are known to have higher efficiency but tend to exhibit higher efficiency roll-off compared with fluorescent organic light-emitting diodes. The efficiency drops at higher luminance occur due to lack of exciton confinement in the emitting layer and exciton quenching induced by high exciton density [3,4].

## 4. Conclusions

Novel host derivatives were prepared during the three-step process procedure by using carbazole and methoxypyridine as building fragments. Values of triplet energy of the derivatives were 2.82 eV for **H1** and 2.81 eV for **H2.** The host materials demonstrate high thermal stability and form amorphous and homogeneous layers with very high glass transition temperatures of 127 °C for **H1** and 139 °C for **H2.** The derivatives were applied as host materials for blue or green PhOLEDs by using blue triplet dopant of iridium(III)[bis(4,6-difluorophenyl)-pyridinato-*N*,*C2*′]picolinate or green dopant of tris(2-phenylpyridine)iridium(III) (Ir(ppy)_3_) as phosphorescent guests. The blue device with 15 wt.% of the FIrpic emitter in host material **H2** exhibited the best overall characteristics, with a current efficiency of 23.9 cd/A, power efficiency of 24.9 lm/W, and high external quantum efficiency of 10.3% at a brightness of 100 cd/m^2^. The most efficient green PhOLED device with 10 wt.% of Ir(ppy)_3_ in host **H2** demonstrated a current efficiency of 33.9 cd/A, power efficiency of 34.1 lm/W, and high external quantum efficiency of 9.4% at high 1000 cd/m^2^ brightness used in technologies of illumination. These properties were demonstrated in non-optimized PhOLEDs, under an ordinary laboratory atmosphere and could be improved in the optimization process. The obtained results confirmed that some of the new host materials are promising components for the development of highly efficient phosphorescent devices.

## Figures and Tables

**Table 1:** Characteristics of devices with host **H2** with Ir(ppy)_3_ and FIrpic dopants.

Device	Dopant	Dopant, wt.%	OV, V	PE, lm/W	CE, cd/A	EQE, %	CIE_xy_ Coordinates	Max Luminance, cd/m^2^
			@100/1000/10,000 cd/m^2^					
I-1	Firpic	15	3.0/3.7/-	24.9/15.8/	23.9/18./-	10.3/8.0/-	(0.18, 0.39)/(0.18, 0.39)/-	9173
I-2	Firpic	17.5	3.3/4.7/-	11.5/6.6/-	12.1/9.8/-	5.1/4.1/-	(0.18, 0.4)/(0.18, 0.4)/-	3317
I-3	Firpic	20	3.2/4.4/-	8.5/5.7/-	8.6/8.0/-	3.6/3.4/-	(0.18, 0.38)/(0.18, 0.38)/-	4377
I-4	Firpic	22.5	2.8/3.6/6.8	5.9/11.5/2.6	5.7/13.1/5.7	2.4/5.5/-	(0.18, 0.4)/(0.18, 0.4)/-	10,350
II-1	Ir(ppy)_3_	7.5	3.2/3.8/5.3	0.8/19.4/19.6	0.9/24.5/32.7	0.3/6.8/9.1	(0.32, 0.62)/(0.33, 0.62)/(0.33, 0.61)	32,390
II-2	Ir(ppy)_3_	10	2.6/3.1/4.3	10/34.1/23.9	10.3/33.9/32.2	3.3/9.4/8.9	(0.34, 0.6)/(0.34, 0.61)/(0.34, 0.61)	38,980
II-3	Ir(ppy)_3_	12.5	3.3/4.1/6.7	18.9/26.2/8.8	20.6/34/18.5	5.7/9.5/7.8	(0.33, 0.61)/(0.33, 0.61)/-	14,170
II-4	Ir(ppy)_3_	15	2.6/3.1/4.3	10.9/34.1/23.9	10.3/33.9/32.2	2.3/7.4/6.9	(0.34, 0.6)/(0.34, 0.61)/(0.34, 0.61)	18,980

## Data Availability

Not applicable.

## Supplementary Materials

The following are available online. Figure SI 1: TGA curve of compound H1. Heating rate: 10 °C/min, Figure SI 2: TGA curve of compound H2. Heating rate: 10 °C/min, Figure SI 3: Photoluminescence and phosphorescence spectra of THF solutions of the materials H1 and H2.

## Sample Availability

Samples are not available from the authors.
