# Supplementary material for: Pyridinyl-Carbazole Fragments Containing Host Materials for Efficient Green and Blue Phosphorescent OLEDs
Source: Molecules. 2021 Jul 30;26(15):4615. doi: 10.3390/molecules26154615 (PMC8348521; doi:10.3390/molecules26154615)
Supplement: Supplementary file 1 [file molecules-26-04615-s001.zip › molecules-1300365-supplementary.pdf]

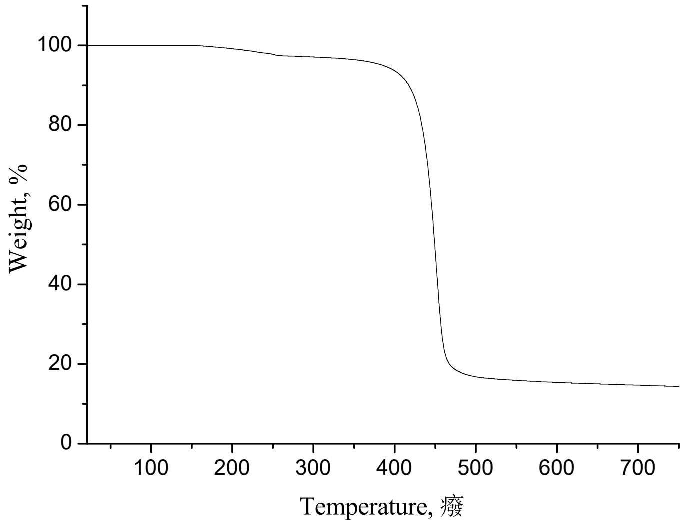

Figure S1. TGA curve of compound **H1**. Heating rate: 10 ºC/min.

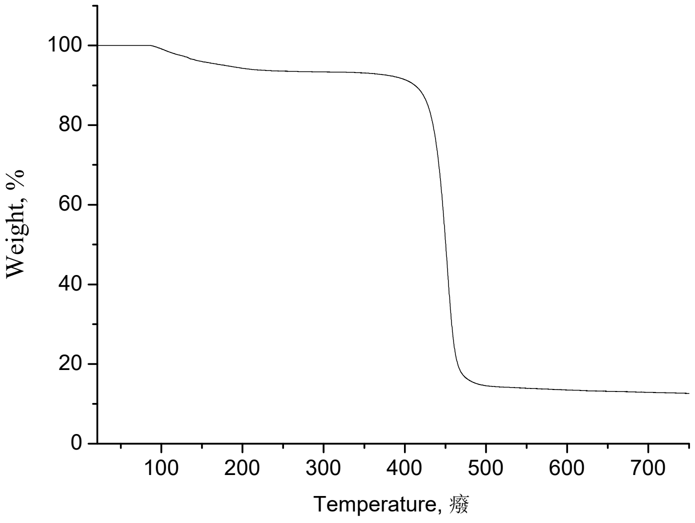

Figure S2. TGA curve of compound **H2**. Heating rate: 10 ºC/min.

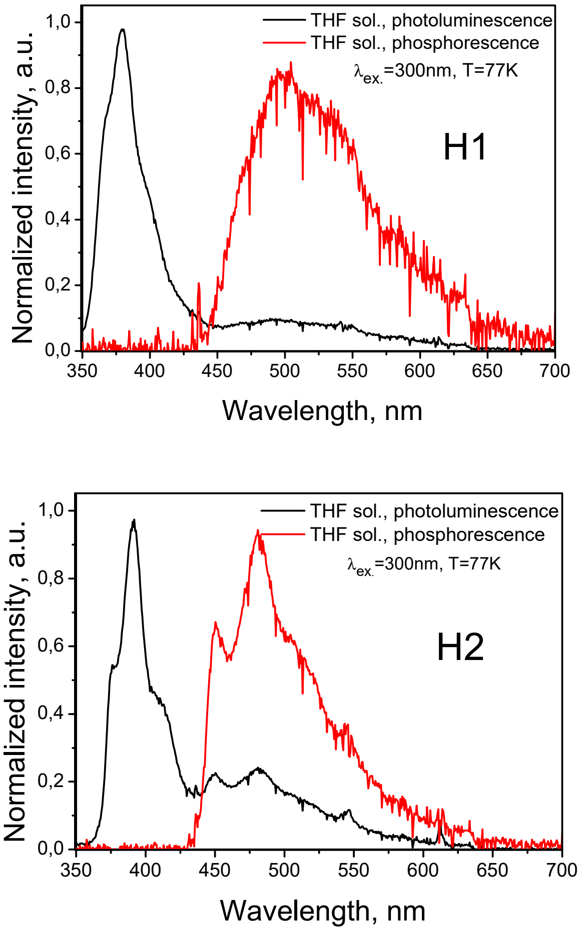

Figure S3. Photoluminescence and phosphorescence spectra of THF solutions of the materials **H1** and **H2**.
